# The Continental Medical Repertory; Exhibiting a Concise View of the Latest Discoveries and Improvements Made on the Continent, in Medicine, Surgery, and Pharmacy

**Published:** 1817-08

**Authors:** 


*The Continental Medical Repertory; exhibiting a concise View of the latest Discoveries and Improvements made on the Continent, in Medicine, Surgery, and Pharmacy.* Conducted by E. VON EMBDEN, M. D. assisted by other Gentlemen of the Faculty. Hamburgh, March 1817. Octavo, pp. 176.

ALTHOUGH a periodical work, yet as printed and published in a *foreign* country, it becomes a legitimate subject of critical analysis in this. We do not fear that we shall be suspected of any envious feelings, when we assert, that this specimen of foreign "discoveries and improvements" is as meagre in matter, and miserable in manner, as any thing of the kind we have ever perused. If Dr. Von Embden and his associates are incapable of constructing a single sentence in English, without violating every rule of grammar and orthography, they ought not to publish in a language of which they are so ignorant. And let us inform those gentlemen, that their work would be more generally perused here, if written in good French than in broken English; for the ears of the profession in England are too much accustomed to elegant composition, not to revolt at the barbarous sentences, and ungrammatical construction, which every where meet the view. We hope these hints will be taken in good part by the Editors, who declare that "they will think themselves highly honoured by any advice, shewing in what manner the work may be improved." *Preface.*—The Repertory is divided somewhat in the manner of our own Journal; viz. into, 1st, An Original Department, containing cases and observations; 2d, An Analytical Department, denominated "Critical Extracts; 3d, Miscellanies; 4th, Medical Intelligence. We shall notice these divisions in the above-mentioned order.

The Original Department contains four papers. 1st. History of a remarkable Convulsive Disorder, by Drs. Rosenstiel and Oberteuffer. The case of this hysterical young lady, none but a German *could* detail; and none but the hapless Reviewer *will* ever peruse! It would be much easier to delineate every step in a very protracted St. Vitus's dance, than enumerate the convulsive twitchings of this young dame during a six months' nervous temperament. The following is the leading symptomatology:

"This young lady, who has been well educated, and is of a most generous and mild disposition, being accompanied hither by her parents, gave me the following account of herself. That for this twelvemonth past she had been seized with frequent despondency, involuntary laughing and crying fits, and slight convulsions; that an insatiable hunger frequently befel her, which obliged her to eat privately several times a day; that in general her feet were cold, and her body bloated, her courses regular, but that she felt herself very much debilitated after them." p. 3.

The other physician's pathological opinion is thus delivered:

"That undoubtedly the highest degree of sensibility and irritability of the whole nervous system might be supposed to exist *in this shocking case,* neither could I contradict its having something hereditary in it. That I further did not consider the cause of the disorder to be idiopathical, but rather a symptomatical one, and that I thought the disorder to be of hypochondriacal origin, and that from over-charging the stomach, as well in quantity as quality, combined with a sedentary life, *infarctus* might have taken place in the *intestinal canal* and abdominal viscera, and have communicated such a disposition to the whole nervous system." p. 6.

*Therapeutics.* ℞. Sacch. alb. ℈j; moschi el. gr. iij. M. fiat pulvis: capiatur ex infus. herb. meliss. 2ndis alternis horis.

℞. Rad. valerian. ℥ß; flor. anthem., flor. aurant., cort. cinchonæ, martis solubilis, āā ʒij; oxyd. zinci ʒß; syr. cort. aurant. q. s. ad consist. elect. Cap. coch. j min. secundis horis.

Embrocations to the abdomen, and assafœtida glysters night and morning.

The above is a sufficient specimen of this Case.

The second paper is on Cancer, by Dr. J. P. Westring. Dr. W's remedy was found out by accident. Having recommended the application of the fresh leaves of marygold to "a most painful carcinomatous induration in the breast," of an elderly lady, "she was enabled to assuage the most burning pain." We shall condense the first Case, as a specimen of Dr. W's specific treatment of Cancer.

CASE. "*A complete Cancer of the Womb,* with violently painful indurations."

Mrs. B. ætat. 47, had schirrus of the womb since the age of 17.

"On the 12th of August, 1812, having for some time suffered much of hæmorrhages, with teazing and burning pains in the hip and groins, terrible colic pains, perpetual eructations, and *such-like* dreadful symptoms, that she thought *it* would choke her. Her face looked bluish grey; she was cold all over, and a *hectic* with a very frequent small pulse had taken place. To complete her misery, a discharge of so fœtid an ichor had come on; that its very smell turned the patient's stomach. An experienced *midwife* having examined her *with terror*, she had found the uterus swollen, *sunk, very painful to the touch, and full of large lumps and deep ulcers,* discharging an ichor of very terrible fœtor."

Having ordered an injection of extr. chærophyllorum, dissolved in decoct. anthem.

"I made her rub in, in the *labia pudenda, a goldsalt,* prepared of a solution of gold in nitric acid, saturated with muriate of ammonia, and precipitated with carbonate of pot-ash. The 12th part of one grain of this precipitate, well triturated with starch, was rubbed in, night and morning, in the place above mentioned."

Under this treatment the patient so far recovered, that,

"—in the beginning of December, the *midwife*, having examined her, declared the womb to be in its normal situation, and to have recovered its natural strength."

In the beginning of the following May,

"—*it appeared, on examination, that she could be considered as perfectly cured.*" p. 28.

On this case of uterine cancer, cured by *goldsalt* rubbed on the labia pudendi, we shall make no comment, as there is not a Tyro in the profession, on this side of the water, who is not quite adequate to form a true judgement.

### CASE. *Lacerated Ileum, without external Marks of Violation.* By Dr. KOPFLI.

J. B. a young man, healthy and strong, received a kick on the belly from a horse, on the 9th of July. Immediately after the accident, he felt a violent pain in the abdomen, extending in all directions. In an hour, nausea, vomiting, oppression on the chest. Cold vinegar fomentations to the abdomen, emollient glysters, diluent drinks. At ten, P. M. great restlessness, pain, nausea, vomiting. Head, hands, and feet, cold; pulse small; thirst. No trace of any contusion could be discovered on the belly, which was neither tense nor swelled. No fæcal evacuation. A vein was opened, but only five ounces of blood could be obtained. Ten drops of laudanum were then given, and the abdomen fomented; glysters continued.

In an hour he recovered a little, the vomiting abated, the pulse expanded, and 15 ounces of blood were abstracted, by which farther relief was obtained.

10th. The vomiting and other distressing symptoms returned: no motion; abdomen swollen; pulse full and strong. V. s. in the foot to a small extent; eight drops of laudanum; fomentations. In the evening, a slight amelioration of the symptoms.

11th. Had rested badly in the night. A febrile paroxysm this morning, with a general perspiration. Pulse irregular, and intermittent; thirst great; occasional inclination to vomit; abdomen more tense, swollen, and painful. Some fæces passed to-day with the glysters.

12th. Much in the same state; except that the abdomen was more puffed, and there was violent pain in the gastric region; the pulse was stronger and more regular; head and hands cold; delirium; burning and pricking sensation in the throat. Another venæsection to eight or nine ounces. This was repeated in the evening, as the blood drawn threw up the inflammatory buff.

13th. Slept in the first part of the night; restless and anxious towards morning. Stercoraceous vomiting, with great gastric pain. The pulse strong and full; eyes sparkling; countenance indicative of great irritation and inflammation. Another bleeding to eight or nine ounces.

14th. Had rested in the beginning of the night as before; thirst not so great this morning; tongue moist, and a little furred; no proper stool; abdomen tense and tender. "Countenance serene and like in health, only that it seemed somewhat disfigured from suffering so much." Two grains of opium were given in divided doses to-day, and he was put into the warm bath, where a general perspiration broke out over the whole body. After remaining three-quarters of an hour, he was taken out and put into a warm bed. He now felt quite easy, and the pulse became perfectly natural; "his looks cheered up by comfort and hope, he felt himself like new-born, and had nothing to complain of."

As may be supposed, this was a deceitful lull before the storm of dissolution. He expired the next day.

*Dissection.* On opening the abdominal cavity, a quantity of thin fœtid liquor mixed with fæces suddenly issued forth. The small intestines were, in several places, inflamed, and glued together. In the ileum, near the abdominal ring, was found a stellated aperture that would admit a nutmeg, and through this the liquor and fæces had passed out into the general cavity. The parietes of the abdomen opposite this were black, and here it is supposed the blow had been received.

Of the Editorial remarks on this case, we shall give our readers a small sample.

"During the progress of the disorder inflammation *came to it*, as well in consequence of the action of the heterogeneous matter as of the violation, which inflammation in consequence of the interruption of continuity, the action of heterogeneous influences, and the strength of the other symptoms, could not bring its usual phænomena to perfection." p. 52.

The fourth and last Article in the Original Department of this Continental Journal, is, "A fatal Case of Phthisis Psoæ, with the Dissection *post mortem*." By Dr. ETMULLER.

J. G. ætat. 59, short stature, and corpulent, had enjoyed constant good health, "excepting an hæmorrhoidal colic." In March last, he was seized with "terrible shiverings," after which, his belly became swelled and his bowels obstructed. The least motion caused him violent pain, eructations, and bilious vomitings. The urine was suppressed; the excrements bloody; the extremities cold; the pulse hard and contracted. Repeated bleedings; the application of fomentations; leeches; embrocations to the abdomen; semicupium; glysters; calomel and opium, gave but momentary relief. In consultation the disorder was pronounced cystitis, the chief pain being seated in the region of the bladder. Digitalis was now added to the former remedies. The symptoms, however, continued unabated, and facies hippocratica with prodromi of mortification appeared. A very copious fœtid stool now came away, and all of a sudden, the pain and other alarming symptoms vanished, leaving only debility behind. A copious discharge also of greasy urine ensued. Calomel combined with digitalis was used for a few days longer, with volatile embrocations to the abdomen. Decoct. cinch. with nutritious diet ordered. Some of the physicians now flattered themselves that they had conquered the disease. Hectic fever, however, came on, and he died in the beginning of August completely emaciated.

*Dissection.* The psoas muscle entirely destroyed by suppuration.

The Second or Analytical Department of the Journal is modestly enough stiled "Critical Extracts," though the Critic might find fault with the title, since it is difficult to say, why the adjective *critical* should be applied to *extracts*. The fact is, that we have not here a series of extracts, but an attempt at analysis, of which attempt it is not our province to judge. We shall merely enumerate the works analysed, and give some slight account of the extracts.

I. " Dr. Chas. Fr. Senff, of Halle, *on the Effects of Hepar Sulphuris in Croup and other Disorders*."

The famous Prize Question of the Ex-Emperor Napoleon, and which has been largely reviewed in the New Med. and Phys. Journal, must have made the English reader sufficiently acquainted with the proposal of employing the abovementioned medicine in croup But, we believe, that it is not likely to meet with much encouragement in this country, while bleeding, blistering, warm bathing, and mercury are so much relied on. The long list of dissimilar diseases in which Dr. S. recommends his favourite remedy, must raise a considerable degree of scepticism in the mind of the reader. Thus, in difficult menstruation, disposition to abortion, hydrencephalus, hepatitis, cardialgia, hooping cough, dysentery, &c. &c. the sulphuret of potash is omnipotent!

II. *Memoire et Rapport sur les Fumigations Sulpheureuses appliquées au Traitement des Affections cutanées et de plusieurs autres Maladies.* Par J. C. Galles, M. D. &c.

Dr. Galles having, for several years, had the charge of certain Parisian hospitals, his opportunities for observing the progress of exanthematic disorders were ample. The unctuous application of sulphur in psora being attended with many inconveniences, Dr. G. set about the construction of an apparatus, by which he might envelope the patient in sulphureous vapour. He first merely used a chafing dish of burning coals, over which sulphur was strewed, and then the dish was put under the blanket while the patient was in bed, carefully securing it. In this manner he employed the fumigations for six or eight months, during which he cured 355 patients affected with itch. Afterwards, however, he constructed a kind of box, by means of which, the vapour was uniformly applied to every part of the patient's body, except the face. Dr. G. affirms, that these fumigations were particularly useful in herpes, and some other cutaneous defœdations, as well as in arthrititis rheumatica, paralysis, scrofula, &c.

III. *Commentationes Societatis Physico-Medicæ apud Universitatem literarum Cæsaream Mosquensem Institutæ.*

J. C. Hillebrandt describes here, a curious dry cupping machine used by the Russians for reposing [reducing] incarcerated herniæ.

"They take a pot capable of holding about a few pounds of liquid, stop up the hole it has got at the bottom with a cork, rarefy the air contained in it by lighted tow, and put it along with the burning tow on the abdomen, previously rubbed with oil or soap. Thus the abdominal parietes and bowels are, not without pain, drawn into the pot, and the parts contained in the rupture into the abdomen. The pot is removed by drawing the cork, and if the effect is not yet complete, the pot is again replaced."

The Author tried this rough popular remedy in many instances, and found it harmless but efficacious.

Next follows a long paper on the plague in Wolhima in the year 1798, by John Minderer; but it conveys no information to the English reader.

A curious case of Lythocele is related by P. F. Pfahler. A man, 31 years of age, (who, when a boy, had two calculi cut out of the scrotum) was again affected with the same complaint in the anterior left part of the scrotum, accompanied by violent pain, dysury, and impotence. An opening being made, three calculi, lying close to the testicle, and weighing one ounce, were extracted. A quantity of fœtid urine flowed from the wound, and with it eleven stones, the size of lentils, were voided. The wound continued open, and the urine to percolate through it. A catheter was now introduced through the natural passage into the bladder, and left in it six weeks, when the wound was perfectly cured, and the patient recovered.

In the next paper, C. J. Haberlein describes the process of preparing the Kumis from mare's milk. This is a popular remedy in Poland and other parts, for pulmonary consumption.

Then follows a description of the Hemitritæus, by Minderer. This is the fever of all low marshy countries, when the sun has caused a certain degree of heat, and the extrication of miasmata has attained its zenith. No pathological or therapeutic information is to be gleaned from this paper. As a specimen of Editorial English, we will adduce the first sentence.

"Without any warning, the patient is taken suddenly with *frost*, whilst inwardly he feels a burning heat." 94.

The next analysis is that of a Nosological and Therapeutical Treatise on the Ophthalmia Catarrhalis Bellica. By FRED. BALTZ, M. D.

This is the Ægyptian or purulent ophthalmia; and after the many able accounts of it which have been published in this country, it would be idle to give any extracts from this jejune performance.

I. The 3d Department, or Miscellanies, opens with a paper on the Non-dilatation and Non-contraction of Arteries in the Pulse; by Professor DOLLINGER.—This gentleman happening to visit some water-works a few years ago, found, that on taking hold of some leaden pipes, in which the waters were forced upwards by pumps, he could plainly perceive an impulse, as often as a wave was driven into them, very much resembling that which is felt in the arteries. Reflecting on this, and remembering that in vivisections he had always found the arteries immoveable,

"He was now anxious to know whether a pulse might be discovered in living animals, in an artery when laid bare. He therefore laid bare the carotid of a dog, in presence of several of his pupils, and a pulse was plainly felt in it (when compressed), tho' no motion was visible in it."

He comes to the conclusion, "that the arteries do not suffer any alternate dilatation or contraction at all, whilst producing that effect called the pulse." 128.

Although the Professor ought to have known that Haller and Bichat came to nearly the same conclusion, yet as his observations coincide with the recent experiments of Parry in this country, and as the subject has undergone an ample discussion in our own and a neighbouring Journal, of late, we shall not here dilate upon it.

II. *Dropsy of the Maxillary Cavity.* A young man laboured under a peculiar dilatation of the antrum maxillare of the right side, which much disfigured his face. It came on gradually, after some wounds received on the head four years previously. The sinus became widened; its parietes enlarged every where, except towards the eye, yielding to pressure with a crackling noise. The nose was pressed towards the left cheek, and the roof of the mouth gave way to pressure. An incision was made along the maxillar margin with the bistoury, and immediately there issued a colourless aqueous fluid without smell. A square piece was removed from the edge of the maxillary bone, and warm wine was injected into the cavity. No pain or swelling came on, and the patient was lost sight of before the aperture closed.

### III. *Remarkable Concretion found in a Tumour.* By Dr. PENADA.

A robust country-woman, 50 years of age, had borne twelve children, and had complained of slight pain in the left hypochondrium ever since her youth. In the month of November, 1806, a tumour became visible, and was treated with emollients. Suppuration ensued, and the wound remained open with callous edges for months. In May 1807, the pain having become more violent, friction was employed, when a roundish body suddenly started out, and flew with violence to some distance. This concretion had the form and size of a hen's egg, externally of a dark slate colour, surface a little rough and granulated, like the pile of velvet. Being divided into two equal parts through its greatest axis, the mass shewed twelve eliptical and concentrical laminæ, each of which was separated from the other by a dark line. The centre contained a white and round nucleus, spongy and chrystalline, consisting again of concentrical laminæ. Professor Melandri, of Padua, analysed it, and says the whole mass of the stone consists of adipocere, modified in the nucleus by a resinous substance.

### IV. *Menstruation through the Mammæ.* Observed by Dr. HINTZE.

A young widow, mother of two children, of a delicate constitution, and strictly moral character, had always menstruated regularly; but catching cold during the catamenial period, her menses stopped, yet without producing any disagreeable effect. At the next menstrual period, the catamenial discharge came from the nipples, without any further change in her health, excepting a transient oppression on the organs of respiration. During many years, the catamenia came vicariously from the breasts, but at length, after bathing in the Altwasser waters, they returned to their normal situation.

V. Bezard relates the case of a woman, who, in thirteen years, was tapped 665 times, while the fluid was 28 times removed by purges and diuretics. On dissection, the peritonæum was found to be three lines in thickness, cartilaginous, and resembling, at first sight, the rind of bacon. The omentum, mesentery, and even the liver, gall-bladder, spleen, pancreas, kidnies, and urinary bladder, had, as it were, disappeared, and a common scirrhous mass was found in their place, towards the right side, resembling the liver in shape, and containing pus.

VI. Dr. Lobstein draws the attention of the medical world to a species of parturient hæmorrhage not yet described, depending on laceration of the interior coat of the vagina. It has as yet been observed only in *primiparæ,* where the unevenness and plaits of the vagina had not yet disappeared, and where, of course there was greater resistance to the child's head. This kind of hæmorrhage, if properly known, is not dangerous, unless the patient be otherwise much enfeebled. The following are the particulars of one of these cases.

After the natural delivery of a primipara, an hæmorrhage took place, which was not very considerable as to quantity, but alarming as to duration. Dr. L. thinking the placenta was loosened in part, removed the afterbirth; but the hæmorrhage continued strong and permanent after the perfect contraction of the womb, and closing of the os tincæ. On more minute examination, a triangular flap, about nine lines long, resembling the caruncula myrtiformis, was found at the posterior and lower part of the vagina, a little above the fossa navicularis, a stream of blood flowing from its point, as if from a vein in the arm. The bleeding immediately ceased on the flap being secured by a ligature.

VII. The following are popular remedies among the Russians in Irkutzk, and in the neighbourhood of the Baikal sea.

1. *Parnassia palustris,* in strangury. 2. *Tanacetum vulgare* in intermittents. 3. Infusion of a species of *Geranium* in suppressio mensium. 4. A species of *Polypodium* in hæmorrhages, particularly uterine. 5. *Chrysantheum leucanthemum* in fluor albus. 6. *Gentiana machrophylla* in delirium and watchfulness. 7. *Dentaria bulbifera* in nervous affections, convulsions, and epilepsy. 8. *Convallaria polygonatum* in gout and rheumatism. 9. *Gentiana campestris* in worms. 10. *Origanum vulgare* in head-ache. 11. *Alisma plantago* in rabies. A poultice made of the herb to the wound, and the powdered root taken internally, but not beyond fifteen or twenty grains at a dose. 12. Nitric acid is used by them in syphilis, and also in fevers. 13. Corrosive sublimate is given in enormous doses in syphilis.

VIII. Dr. Harles, of Erlangen, recommends a watery solution of sulphate of zinc for the cure of scabies: it is generally cured in eight or ten days. From ʒj to ʒij zinci sulph. is dissolved in from an ounce and a half to two ounces of decoct. ulmi, and used as a lotion twice or thrice a-day.

From the 4th Department, entitled, "Medical Intelligence," we do not find any thing that is particularly worthy of notice, and therefore shall close our analysis.

We flatter ourselves that we are too much actuated by a love of science, and of the profession, to harbour a jealous feeling towards a rival contemporary. We should not grieve to see a new candidate start every year, since competition is the grand stimulus to exertion, and forms the crucible by which the sterling ore must sooner or later be separated from the dross. Whatever figure the "Continental Medical Repertory" may make in the meridian of Germany, we venture to affirm, that without a total revolution in its constitution, it will not survive the first *volume*, if a second *number*, indeed, should ever see the light. It is, in fact, so far beneath criticism, that, in pity towards its misguided Editors, we have only exhibited its merits, and drawn almost a total veil over its innumerable absurdities. In justice to our readers, however, we cannot avoid apprizing them of what they are to expect in the Continental Medical Repertory.

